# Environmental Smoking and Smoking Onset in Adolescence: The Role of Dopamine-Related Genes. Findings from Two Longitudinal Studies

**DOI:** 10.1371/journal.pone.0086497

**Published:** 2014-01-21

**Authors:** Marieke Hiemstra, Marloes Kleinjan, Onno C. P. van Schayck, Rutger C. M. E. Engels, Roy Otten

**Affiliations:** 1 Behavioural Science Institute, Radboud University, Nijmegen, The Netherlands; 2 Care and Public Health Research Institute (CAPHRI), Maastricht University, Maastricht, The Netherlands; Yale University, United States of America

## Abstract

Although environmental smoking (i.e., paternal and maternal smoking, sibling smoking, and peer smoking) is one of the most important factors for explaining adolescent smoking behavior, not all adolescents are similarly affected. The extent to which individuals are vulnerable to smoking in their environment might depend on genetic factors. The aim of this study was to examine the interplay between environmental smoking and genes encoding components of the dopaminergic system (i.e., dopamine receptor *D2*, *D4*, and dopamine transporter *DAT1*) in adolescent smoking onset. Data from two longitudinal studies were used. *Study 1* consisted of 991 non-smoking early adolescents (mean age = 12.52, SD = .57) whereas *study 2* consisted of 365 non-smoking middle to late adolescents (mean age = 14.16, SD = 1.07) who were followed for 16 and 48 months, respectively. Logistic regression analyses were conducted using Mplus. In *study 1*, we found positive associations between parents' and friends' smoking at the first measurement and smoking status 16 months later. In *study 2* we found a positive association between friends' smoking and smoking onset 48 months later. Neither study demonstrated any interaction effects of the *DRD2*, *DRD4*, or *DAT1* genotypes. In conclusion, the effects of environmental smoking on smoking onset are similar for adolescent carriers and non-carriers of these specific genes related to the dopaminergic system.

## Introduction

Despite the disturbing consequence of tobacco use, thousands of young people start smoking each day [1]. Several studies have shown that environmental factors (e.g., parental, sibling, and peer smoking) are consistent predictors of juvenile smoking. A recent meta-analysis [2] of 58 studies revealed that parental and sibling smoking increased the risk for child and adolescent smoking. Children whose parents both smoke are almost three times more likely to start smoking, and smoking by a sibling doubles the risk of adolescent smoking [2]. Regarding peer influences, reviews showed a strong association between friends' smoking and adolescent smoking. Adolescents with smoking friends are more likely to smoke than those with only non-smoking friends [3]–[5].

Yet adolescents are not identically affected by their environment [6]. Genetic factors might underlie inter-individual differences in the susceptibility to environmental smoking, suggesting possible gene-environment interactions [7]. The combination of specific genotypes and social contexts might trigger certain phenotypes (e.g., smoking initiation) [8].

Using twin data, White and colleagues [9] showed that heritability factors affect adolescent smoking through an effect on the choice of friends. Another twin study showed that adolescents who are genetically predisposed to start smoking are also more susceptible to best friends' smoking [10]. These twin studies suggest an interaction between environmental smoking behavior and genetic factors, but they failed to provide information on the specific genes involved. For parental and sibling smoking, no previous behavioral genetic interaction studies on smoking were found.

Molecular genetic studies on smoking have focused on the genes encoding components of the dopaminergic system as nicotine increases dopaminergic activity in the brain, thereby resulting in feelings of pleasure or reward [11]. Important functions of the dopaminergic system are the activation of postsynaptic receptor neurons (i.e., dopamine receptors) and dopamine reuptake by presynaptic neurons (i.e., dopamine transporters). Candidate genes involved in dopaminergic neurotransmission are the dopamine receptor *D2* and *D4* and the dopamine transporter gene *DAT1* [12]. Review studies and meta-analyses on the direct relation of dopaminergic system on smoking initiation showed mixed results. For the *DRD2* genotype, a weak association was found between *DRD2* and adolescent smoking initiation [13], [14]. In a more recent meta-analysis no association with smoking initiation was found for the overall sample. However, when the sample was split up for different ethnic backgrounds, an association was found for Caucasians but not for Asians [15]. For the *DRD4* [16]–[18] and *DAT1* genotypes [13], [19], [20], some studies showed a positive association whereas others did not. It has been suggested that genetics is likely to play a direct and profound role in more persistent and progressive stages of smoking, when the sensitization of dopaminergic pathways (through repetitive exposure of nicotine) has occurred [21]. Although behavioral genetic studies have supported this assumption [9], [22], [23], a recent review of molecular studies concentrating on the effects of a specific polymorphism in the dopaminergic system (i.e., DRD2) found no effects or mixed effects on progressive stages of smoking [14]. Hence, the supposed direct link between genes related to the dopaminergic system and smoking is not well-established.

The lack of consistent evidence for direct effects of genetic polymorphisms on smoking onset does not rule out the idea that dopamine genes relate to smoking initiation indirectly through an increased susceptibility to environmental factors. Inter-individual genetic variation might cause people to react differently to environmental smoking. To our knowledge, no molecular interaction studies between environmental smoking and genes related to the dopaminergic system on smoking initiation have been conducted. This study will fill this gap by concentrating on the environment and genetic effects as well as their interplay on smoking initiation by means of a longitudinal design. Based on the literature, we expect environmental smoking to affect genetically predisposed children to start smoking. There are two potentials pathways through which these effects might operate. First, it could be that some children are more susceptible to rewards in general. Previous research has demonstrated that children with smoking parents react stronger to smoking-related cues than children of non-smoking parents (even if they have never smoked) [24], indicating that children of smoking parents develop automatic cognitive responses in the form of attention toward smoking. The *DRD4* risk allele has been found to be related to attentional bias [25], [26]. We expect carriers of the *DRD4*-risk allele to be more sensitive for an attentional bias to smoking and subsequent smoking initiation when exposed to smoking behavior in their environment. Second, molecular genetic studies have shown that the dopaminergic system is related to novelty seeking (*DRD4*: [16], [27] ; *DRD2:* [28]–[30]) and impulsivity (*DRD4:* [27] ; *DRD2*: [31] ; *DAT1*: [30]). Therefore, some risk allele carriers might be more likely to show elevated levels of novelty seeking or impulsivity and in turn be more likely to start smoking when in a context with people who smoke and cigarettes are readily available.

The current study examined the interactions between environmental smoking (e.g., parental smoking, sibling smoking, and smoking by peers) and the dopamine receptor genes *DRD2* and *DRD4* as well as the dopamine transporter gene *DAT1* on the relationship with smoking initiation. Two independent longitudinal data sets were used to allow for a replication of the results, which is essential to gain insights into the consistency of findings [32].

## Methods

### Procedure

#### Study 1

Data were derived from a longitudinal study focusing on genetic and environmental influences on substance use among Dutch adolescents. Schools in the Eastern and Southern part of the Netherlands were sent study information and were then called and asked to participate. Twenty-two schools agreed to participate. In 2010, the principal investigator (MK) visited every school to provide all children in the first year of high school with information on the study. Children who wanted to participate were asked to provide a consent form that they had signed as well as at least one of their parents/guardians. In total, 1,399 adolescents were recruited. During each wave, the participants completed an online or paper-and-pencil questionnaire during school hours. Students were explicitly instructed that all questions were about their regular patterns, unless otherwise stated. The study consisted of five waves (T1 through T5), and 1,360 (T1), 1,230 (T2), 1,183 (T3), 1,188 (T4), and 1,099 (T5) adolescents participated in each wave (response rate of 78.1% across all five waves). Time intervals between the waves were approximately four months. At T1, saliva samples were collected for DNA extraction (Oragene, DNA Genotek Inc.). Due to limited financial resources, 1,210 adolescents were genotyped at T1; 4 participants could not be genotyped. The design for this study was evaluated and ethically approved by an independent medical ethical committee (Medical Ethical review committee for Mental Health (METiGG), Utrecht, The Netherlands). At the end of the study, all participants received a small gift, and at each wave gift certificates were raffled.

### Sample characteristics

#### Study 1

At baseline, 991 never smoking adolescents were selected. Table 1 shows the baseline characteristics. Logistic regression analysis showed that non-selected genotyped smokers (*n* = 200), compared to selected genotyped non-smokers (*n* = 991), were less likely to be girls (*OR* = .60, *95%CI* = .40–.89, *p* = .01), were less educated (*OR* = .89, *95%CI* = .81–.98, *p* = .02), and had more smoking friends (*OR* = 2.86, *95%CI*  = 1.85–4.44, *p*<.001), smoking best friends (*OR* = 2.39, *95% CI* = 1.79–3.21, *p*<.001), and smoking siblings (*OR* = 2.65, *95% CI* = 1.64–4.27, *p*<.001).

**Table 1:** Baseline Characteristics of *Study 1* and *Study 2*.

	*Study 1 (n = 991)*	*Study 2 (n = 365)*
Gender		
Boys	465 (46.9)	175 (47.9)
Girls	526 (53.1)	190 (52.1)
Age (mean (SD))	12.52 (.57) (11–15)	14.16 (1.07) (13–16)
Ethnicity		
Dutch	958 (96.7)	348 (98)
Other	33 (3.3)	7 (2)
Educational level*		
Low	535 (53.9)	80 (22.2)
Middle	312 (31.5)	148 (41.1)
High	143 (14.5)	131 (36.4)
Adolescent smoking T2		
Smoker	55 (6.3)	58 (16.1)
Non-smoker	821 (93.7)	302 (83.9)
Adolescent smoking T3		
Smoker	89 (10.7)	87 (24.2)
Non-smoker	744 (89.3)	272 (75.8)
Adolescent smoking T4		
Smoker	117 (14.1)	128 (35.7)
Non-smoker	715 (85.9)	231 (64.3)
Adolescent smoking T5		
Smoker	140 (17.9)	127 (39.1)
Non-smoker	641 (82.1)	198 (60.9)
Smoking mother T1		
Never smoked	570 (58.4)	103 (28.3)
Former smoker	242 (24.8)	195 (53.6)
Current smoker	164 (16.8)	66 (18.1)
Smoking father T1		
Never smoked	462 (47.9)	95 (26.4)
Former smoker	268 (27.8)	194 (53.9)
Current smoker	235 (24.4)	71 (19.7)
Smoking sibling T1		
Having no smoking sibling(s)	818 (90.5)	-
Having one or more smoking sibling(s)	86 (9.5)	-
Never smoked	-	272 (74.7)
Former smoker	-	66 (18.1)
Current smoker	-	26 (7.1)
Smoking friends T1		
Having no smoking friends'	786 (79.3)	192 (52.9)
Having smoking friends'	205 (20.7)	171 (47.1)
Smoking best friend T1		
Never smoked	936 (94.5)	254 (72.8)
Former smoker	18 (1.8)	72 (20.6)
Current smoker	37 (3.7)	23 (6.6)
*DRD2*		
Non-risk (A2A2)	633 (64.1)	254 (69.8)
Risk (A1A2/A1A1)	355 (35.9)	110 (30.2)
*DRD4*		
Non-risk (<7 repeats)	620 (63.3)	215 (59.2)
Risk (≥7 repeats)	360 (36.7)	148 (40.8)
*DAT1*		
Non-risk (8/10, 10/10, 10/11)	578 (58.6)	209 (58.2)
Risk (9/9, 9/10, 9/11)	408 (41.4)	150 (41.8)

Values are numbers (percentage) unless stated otherwise.

*Note.*

* Low = preparatory school for technical and vocational training, Middle = intermediate or general education, high = preparatory college and university education.

### Procedure

#### Study 2

Data were used from five yearly waves of the longitudinal Dutch “Family and Health” study [33], [34]. Addresses of 5,062 families consisting of a father, a mother, and two adolescents aged 13 to 16 years were selected from 22 municipality registers. A letter was sent to these families, asking them to participate in this study. In total, 885 families were interested and gave written informed consent. From these families, 765 met the inclusion criteria (i.e., parents were married or were living together and all family members had to be biologically related). Due to limited financial resources, a further selection was made of 428 families to obtain an equal division of education and number of sibling dyads (i.e., 108 boy–boy, 118 boy–girl, 106 girl–girl, and 96 girl–boy).

An interviewer visited the families in their homes between November 2002 and April 2003 (T1). During these visits, each family member was asked to complete a questionnaire. Respondents were asked to sit separately and not talk to one another about the questions to ensure anonymity. Attrition was low. The number of participating families was 416 (T2), 404 (T3), 356 (T4), and 326 (T5), which is a response rate of 76% across all five waves. If all four family members filled out the questionnaire, the family received €30 per wave. At T4, DNA samples were collected by means of saliva (Oragene; DNA Genotek Inc., Ottowa, ON, Canada). Three hundred eleven families agreed to provide genetic data. Parental written consent was obtained for all participating adolescents, and the research design for this study was approved by the independent medical ethics committee METiGG in Utrecht, the Netherlands (research 6209).

### Sample characteristics

#### Study 2

At baseline, 365 never smoking adolescents were selected (165 older and 200 younger adolescent siblings). Table 1 shows the baseline characteristics. Logistic regression analysis indicated that, compared to genotyped non-smokers (*n* = 365), adolescents who were genotyped smokers (*n* = 254) were older (*OR* = 1.26, *95%CI* = 1.03–1.53, *p = *.02), and more likely to have smoking friends T1 (*OR* = 1.97, *95%CI* = 1.26–3.07, *p = *.003), smoking best friends (*OR* = 3.31, *95%CI* = 2.44–4.48, *p*<.001), and smoking siblings (*OR* = 3.11, *95%CI* = 2.27–4.25, *p*<.001).

### Measures

#### Adolescent smoking

At each wave, the adolescents indicated, on a nine-point scale, which stage of smoking applied to them. Response categories ranged from 1 = “I have never smoked, not even one puff” to 9 “I smoke at least once a day” [35]. For logistic regression analyses, these responses were recoded to non-smoker = 0 (never smoking) and smoker = 1 (any experience with lifetime smoking) (cf. [33]).

#### Paternal and maternal smoking

In *study 1*, adolescents used an eight-point scale to indicate which stage of smoking applied to their parents. Response categories ranged from 1 = “My father/mother have never smoked” to 8 = “My father/mother smokes more than 31 cigarettes a day”. In *study 2*, both parents were asked to report which stage of smoking applied to them using the same scale as the adolescents [35]. One of the nine responses was less appropriate for adults (i.e., “I have tried smoking once in a while”) and was omitted (cf., [33]). In both studies, the answers were recoded into three categories: 1 = “never smoked”, 2 = “former smoker”, and 3 = “current smoker” (cf. [36]).

#### Sibling smoking

In *study 1* adolescents were asked two questions related to how many of their siblings were smoking. Response categories ranged from 0 = “None of my brothers/sisters smokes” to 4 = “Four of my brothers/sisters smoke”. The answers of both questions were summed up and dichotomized into 0 = “Having no smoking sibling(s)” and 1 = “Having one or more smoking sibling(s)”. In *study 2*, the same question the target adolescent was asked for their sibling [37], and the responses were recoded into three categories: 1 = “never smoked”, 2 = “former smoker”, and 3 = “current smoker” (cf. [36]).

#### Friends' smoking

In both studies, adolescents were asked whether their friends smoked: “How many of your friends smoke?” Response options were 1 = “no one”, 2 = “less than half”, 3 = “half”, 4 = “more than half”, and 5 = “all of them”. Answers were dichotomized into 0 = “Having no smoking friends” and 1 = “Having smoking friends” [38].

#### Best friends' smoking

In *study 1* the same question about the maternal or paternal smoking behavior was asked about best friends. In *study 2* respondents were asked to indicate on an eight-point scale which stage of smoking applied to their best friends [37]. Responses ranged from 1 = “My best friend has never smoked, not even one puff” to 8 = “My best friend smokes at least once a day”. In both studies the answers were recoded into three categories: 1 = “never smoked”, 2 = “former smoker”, and 3 = “current smoker” (cf. [36]).

### Genotyping

#### Study 1

*DRD2*. For the *DRD2* TaqI A C >T polymorphism (rs1800497) ready-made Taqman Allelic Discrimination assays were ordered (Taqman Allelic Discrimination ID: DRD2 (rs1800497), C_7486676_10, reporter 1: VIC-A-Allele, forward assay). Genotyping for the polymorphisms DRD2 (rs1800497) was carried out in a volume of 5 µl containing 10 ng of genomic DNA, 1× Taqman Mastermix (2×; Applied Biosytems, Nieuwerkerk a/d Ijssel, The Netherlands) and 0.5× Taqman assay (40×). Each amplification for the Taqman Allelic Discrimination assays C_7486676_10 and C_8950074_1_ was performed by an initial denaturation at 95°C for 12 minutes, followed by 40 cycles of denaturation at 92°C for 15 seconds and annealing/extension at 60°C for 1 minute; this was carried out on a 7500 Fast Real-Time PCR System. Genotypes were scored using the algorithm and software supplied by the manufacturer (Applied Biosystems). Hardy–Weinberg equilibrium (HWE) proportions were estimated from the parental genotype information. No deviations from HWE were detected (*p* = .71). To maximize the power, the *DRD2* genotype was dummy coded into 1 non-risk (A2A2) and 2 risk (A1A2 and A1A1) (cf. [14]).

*DRD4.* The 48-base-pair direct repeat polymorphism in *DRD4* was genotyped with PCR on 10 ng genomic DNA using 0.5 µM fluorescently labeled forward primer (VIC-5′-GCGACTACGTGGTCTACTCG-3′) and reverse primer (5′-AGGACCCTCATGGCCTTG-3′), 1× GC buffer I TaKaRa (Westburg, Leusden, The Netherlands), 0.4 mM of dNTPs TaKaRa (Westburg, Leusden, The Netherlands), 1 M of Betaïne, and 0.05 U of TaKaRa LA Taq (Westburg, Leusden, The Netherlands). The cycling conditions for the polymerase chain reaction started with 1 minute at 94°C, followed by 35 cycles of 30 seconds at 94°C, 30 seconds at the optimized annealing temperature (58°C), and 1 minute at 72°C, then followed by an extra 5 minutes at 72°C. The product of the amplification was diluted in H_2_O at a ratio of 1∶1. HWE proportions were estimated, and no deviations from these proportions were found (*p* = .53). The *DRD4* genotype was dummy-coded into two categories: 1 non-risk (short allele, fewer than 7 repeats) and 2 risk (7-repeat allele carriers (or more), at least one long allele) (cf. [16]).

*DAT1*. The 40-base-pair VNTR in the *SLC6A3* (*DAT1*) gene was genotyped with 30 ng Genomic DNA as the template. PCR was performed with 1× AmpliTaq Gold® 360 Master Mix (Life Technologies) and 0.33 mM of fluorescently labeled forward primer (NED- 5′- TGTGGTGTAGGGAACGGCCTGAG-3′) and reverse primer (5′-CTTCCTGGAGGTCACGGCTCAAGG-3′) in a total volume of 15 µl. Amplification was performed using the following protocol: 95°C for 10 minutes followed by 35 cycles of denaturation for 30 seconds at 95°C, 30 seconds of annealing at 58°C, and primer extension at 72°C for 1 minute, followed by a final extension at 72°C for 10 minutes. The product of the amplification was diluted in H_2_O at a ratio of 1∶1. HWE proportions were estimated, and no deviations from these proportions were found (*p* = .40). The *DAT1* genotype was dummy coded into 1 non-risk (8/10, 10/10, and 10/11) and 2 risk (9/9, 9/10, and 9/11) (cf. [39]).

#### Study 2

*DRD2*, *DRD4*, and *DAT1* were genotyped in almost the same manner as in *study 1*. HWE proportions were estimated, and no deviations were found (*p* = .12 for *DRD2*, *p* = .87 for *DRD4*, *p* = .40 for *DAT1*). See also [34].

### Analyses

Descriptive statistics for *study 1* and *study 2* were calculated using SPSS 19.0. Logistic regression analyses in Mplus [40] were used to examine the associations between environmental smoking and smoking onset as well as the moderating role of the specific dopamine genotypes in this relationship. For every separate combination of environmental smoking factors and dopamine genotypes, logistic regression analyses were conducted, resulting in a total of 15 for *study 1* and 15 for *study 2*. In a first step, we tested whether potentially important covariates (i.e., age, gender, education) were related to smoking status at T5. In the second step, the environmental smoking at T1 (i.e., paternal and maternal smoking, sibling smoking, or (best) friend(s′) smoking, respectively) and the dopamine genotype (i.e., *DRD2*, *DRD4*, or *DAT1*, respectively) were added to the model. In the third step, the interaction terms between the specific environmental smoking variable and the dopamine gene of interest were entered.

Data from *study 1* were nested within schools (*N* = 22) while data from *study 2* were nested within families (i.e., oldest and youngest siblings (*N* = 428)). To correct the standard errors of the parameters estimated for dependency, the CLUSTER command in combination with the TYPE = COMPLEX procedure in Mplus was used [40]. The parameters in the model were estimated using the Maximum Likelihood estimator with robust stand errors (MLR) as smoking onset was skewed. Due to the number of tests, we used a Bonferroni correction. Results were considered significant if the *p*-value was ≤.003.

## Results

### Descriptives and correlations

Table 2 shows the Pearson's correlations of the model variables. In *study 1* we found that maternal and paternal smoking at T1 was positively related to adolescent smoking at T2 through T5. For sibling smoking at T1, only a positive relation with adolescent smoking at T2 was found. For smoking behavior of friends and best friends, a positive relation with adolescent smoking at T2 through T5 was found. No associations between *DRD2*, *DRD4*, and *DAT1* genotypes and adolescent or environmental smoking were found.

**Table 2:** *P*earson's Correlations among the study variables of *study 1* and *study 2*.

	*Range*	*1.*	*2.*	*3.*	*4.*	*5.*	*6.*	*7.*	*8.*	*9.*	*10.*	*11.*	*12.*
*1. Adolescent smoking T2*	0–1	*-*	.58***	.53***	.42***	.11*	.08	.11*	.23***	.20***	−.08	−.06	−.002
*2. Adolescent smoking T3*	0–1	.54***	-	.67***	.57***	.10	.10	.12*	.21***	.07	−.03	.01	.04
*3. Adolescent smoking T4*	0–1	41***	.58**	-	.70***	.12*	.10	.13*	.14***	.02	−.04	−.01	−.01
*4. Adolescent smoking T5*	0–1	.37***	.48**	.61**	-	.04	.05	.13*	.14*	.03	−.06	.06	−.004
*5. Smoking mother T1*	0–2	.10**	.17**	.07*	.14**	-	.24***	.05	.05	.10	.04	−.05	−.11*
*6. Smoking father T1*	0–2	.10**	.12**	.12**	.11**	.29**	-	.10	.01	.10	.02	.04	−.12*
*7. Smoking sibling T1*	0–2	.12**	.06	.03	.07	.16**	.17**	-	.04	.01	−.04	.01	−.10
*8. Smoking friends T1*	0–1	.20***	.22**	.23**	.17**	.09**	.15**	.08*	-	.26***	−.03	−.08	−.01
*9. Smoking best friend T1*	0–2	.16***	.16**	.13**	.12**	.01	.06	.05	.38**	-	−.02	−.03	−.06
*10. DRD2*	1–2	−.02	−.03	.01	.04	.03	.02	−.01	−.002	−.02	-	.02	.08
*11. DRD4*	1–2	.004	.02	.06	.05	.03	.02	−.04	.01	.004	−.004	-	−.11***
*12. DAT1*	1–2	.001	.01	.02	−.003	.01	−.05	.02	.02	.00	.02	−.03	-

*Note.* Correlations for *study 1* can be found under the diagonal and for *study 2* above the diagonal;

* *p*<.05,

** *p*<.01,

*** *p*<.001.

In *study 2*, maternal smoking at T1 was positively associated with adolescent smoking at T2 and T4. For paternal smoking at T1, no association with smoking onset was found. Sibling smoking and friends' smoking at T1 were both positively correlated with adolescent smoking at T2 to T5. Best friends' smoking at T1 was positively associated with adolescent smoking at T2. Paternal and maternal smoking behavior at T1 were negatively associated with the *DAT1*, indicating that the risk *DAT1* genotype was associated with lower levels of parental smoking.

### Logistic regression analyses

#### Study 1

Table 3 and 4 show the results of the fifteen logistic regression analyses on family and friends smoking. In step 1, we found that higher levels of education were associated with lower likelihoods for smoking onset at T5. In step 2, we found that maternal and friends' smoking at T1 was positively related to adolescent smoking onset at T5. A significant direct effect of *DRD4* was found in the analysis corrected for sibling smoking. For *DRD2* and *DAT1*, no direct effects on smoking onset were found. In step 3, no significant interactions were found between the environmental smoking variables and the *DRD2*, *DRD4*, or *DAT1* genotypes.

**Table 3:** Logistic regression analyses family smoking at T1 predicting smoking onset at T5 and the moderating role of *DRD2*, *DRD4* and *DAT1* genotypes for *study 1*.

	*Maternal smoking*			*Paternal smoking*			*Sibling smoking*		
	*DRD2*	*DRD4*	*DAT1*	*DRD2*	*DRD4*	*DAT1*	*DRD2*	*DRD4*	*DAT1*
	OR _(95% CI)_	OR _(95% CI)_	OR _(95% CI)_	OR _(95% CI)_	OR _(95% CI)_	OR _(95% CI)_	OR _(95% CI)_	OR _(95% CI)_	OR _(95% CI)_
*Step 1*									
*Age*	1.11 _(0.83–1.48)_ *p* = .49	1.11 _(0.83–1.48)_ *p* = .49	1.11 _(0.83–1.48)_ *p* = .49	1.11 _(0.83–1.48)_ *p* = .49	1.11 _(0.83–1.48)_ *p* = .49	1.11 _(0.83–1.48)_ *p* = .49	1.11 _(0.83–1.48)_ *p* = .49	1.11 _(0.83–1.48)_ *p* = .49	1.11 _(0.83–1.48)_ *p* = .49
*Gender*	1.01 _(0.79–1.28)_ *p* = .94	1.01 _(0.79–1.28)_ *p* = .94	1.01 _(0.79–1.28)_ *p* = .94	1.01 _(0.79–1.28)_ *p* = .94	1.01 _(0.79–1.28)_ *p* = .94	1.01 _(0.79–1.28)_ *p* = .94	1.01 _(0.79–1.28)_ *p* = .94	1.01 _(0.79–1.28)_ *p* = .94	1.01 _(0.79–1.28)_ *p* = .94
*Education*	.84 _(0.75–0.93)_ *p = *.001	.84 _(0.75–0.93)_ *p = *.001	.84 _(0.75–0.93)_ *p = *.001	.84 _(0.75–0.93)_ *p = *.001	.84 _(0.75–0.93)_ *p = *.001	.84 _(0.75–0.93)_ *p = *.001	.84 _(0.75–0.93)_ *p = *.001	.84 _(0.75–0.93)_ *p = *.001	.84 _(0.75–0.93)_ *p = *.001
*Step 2*									
*Environmental smoking T1*	1.56 _(1.22–1.99)_ *p = *.000	1.52 _(1.18–1.95)_ *p* = .001	1.54 _(1.21–1.97)_ *p = *.000	1.36 _(1.08–1.70)_ *p = *.008	1.35 _(1.07–1.71)_ *p* = .01	1.35 _(1.07–1.70)_ *p = *.01	1.51 _(.81–2.81)_ *p = *.19	1.59 _(.86–2.92)_ *p = *.14	1.61 _(.90–2.91)_ *p = *.11
*Genotype*	1.15 _(.83–1.61)_ *p = *.41	1.30 _(.95–1.79)_ *p* = .10	0.96 _(.64–1.43)_ *p = *.82	1.14 _(.81–1.60)_ *p = *.45	1.34 _(.98– 1.84)_ *p = *.06	1.01 _(.71–1.43)_ *p = *.97	1.10 _(.75–1.61)_ *p* = .62	1.45 _(1.15–1.84)_ *p* = .001	.96_(.66–1.39)_ *p = *.81
*Step 3*									
Genotype*environmental smoking	0.58 _(.35–.98)_ *p = *.04	.97 _(.59–1.58)_ *p = *.89	1.34 _(.94–1.92)_ *p = *.10	.93 _(.51–1.70)_ *p = *.81	.72 _(.42–1.26)_ *p = *.25	.95 _(.57–1.60)_ *p = *.86	.89_(.34–2.32)_ *p* = .80	2.34 _(.51–10.82)_ *p = *.27	1.45 _(.57–3.71)_ *p = *.44

*Note*. gender: 1 = boy, 2 = girl; OR = Odds Ratio; 95% CI = 95% Confidence Interval.

**Table 4:** Logistic regression analyses friends smoking at T1 predicting smoking onset at T5 and the moderating role of *DRD2*, *DRD4* and *DAT1* genotypes for *study 1*.

	*Friends smoking*			*Best friends smoking*		
	*DRD2*	*DRD4*	*DAT1*	*DRD2*	*DRD4*	*DAT1*
	OR _(95% CI)_	OR _(95% CI)_	OR _(95% CI)_	OR _(95% CI)_	OR _(95% CI)_	OR _(95% CI)_
*Step 1*						
*Age*	1.11 _(.83–1.48)_ *p* = .49	1.11 _(.83–1.48)_ *p* = .49	1.11 _(.83–1.48)_ *p* = .49	1.11 _(.83–1.48)_ *p* = .49	1.11 _(.83–1.48)_ *p* = .49	1.11 _(.83–1.48)_ *p* = .49
*Gender*	1.01 _(.79–1.28)_ *p = *.94	1.01 _(.79–1.28)_ *p = *.94	1.01 _(.79–1.28)_ *p = *.94	1.01 _(.79–1.28)_ *p = *.94	1.01 _(.79–1.28)_ *p = *.94	1.01 _(.79–1.28)_ *p = *.94
*Education*	.84 _(.75–.93)_ *p = *.001	.84 _(.75–.93)_ *p = *.001	.84 _(.75–.93)_ *p = *.001	.84 _(.75–.93)_ *p = *.001	.84 _(.75–.93)_ *p = *.001	.84 _(.75–.93)_ *p = *.001
*Step 2*						
*Environmental smoking*	2.44 _(1.53–3.91)_ *p* = .000	2.30 _(1.42–4.32)_ *p* = .001	2.39 _(1.48–3.84)_ *p* = .000	1.64 _(1.01–2.65)_ *p* = .05	1.60_(.94–2.72)_ *p* = .08	1.62_(1.00–2.62)_ *p* = .05
*Genotype*	1.14_(.81–1.60)_ *p = *.45	1.37 _(1.01–1.87)_ *p = *.04	1.00 _(.71–1.40)_ *p = *.99	1.17 _(.82–1.66)_ *p = *.39	1.41,_ (1.05–1.89)_ *p = *.02	1.01_(.72–1.41)_ *p* = .97
*Step 3*						
Genotype* environmental smoking	1.05 _(.59–1.87)_ *p*_ = _.88	.58 _(.32–1.02)_ *p = *.06	.86 _(.29–2.56)_ *p = *.79	.99_(.44–2.22)_ *p = *.97	.91_ (.39–2.10)_ *p = *.82	1.18 _(.58–2.41)_ *p = *.64

*Note*. gender: 1 = boy, 2 = girl; OR = Odds Ratio; 95% CI = 95% Confidence Interval.

#### Study 2

Table 5 and 6 show the results of *study 2*. In step 1, no significant effects were found for age, gender, or education. In step 2, we found a significant trend of friends' and sibling smoking on adolescent smoking. No significant effects were found for paternal, maternal, and best friends' smoking. Moreover, no significant effects were found for the dopamine genotypes. Finally, in step 3, no significant interaction effects were found.

**Table 5:** Logistic regression analyses family smoking at T1 predicting smoking onset at T5 and the moderating role of *DRD2*, *DRD4*, and *DAT1* genotypes for *study 2*.

	*Maternal smoking*			*Paternal smoking*			*Sibling smoking*		
	*DRD2*	*DRD4*	*DAT1*	*DRD2*	*DRD4*	*DAT1*	*DRD2*	*DRD4*	*DAT1*
	OR _(95% CI)_	OR _(95% CI)_	OR _(95% CI)_	OR _(95% CI)_	OR _(95% CI)_	OR _(95% CI)_	OR _(95% CI)_	OR _(95% CI)_	OR _(95% CI)_
*Step 1*									
*Age*	.92 _(.75–1.12)_ *p* = .40	.92 _(.75–1.12)_ *p* = .40	.92 _(.75–1.12)_ *p* = .40	.92 _(.75–1.12)_ *p* = .40	.92 _(.75–1.12)_ *p* = .40	.92 _(.75–1.12)_ *p* = .40	.92 _(.75–1.12)_ *p* = .40	.92 _(.75–1.12)_ *p* = .40	.92 _(.75–1.12)_ *p* = .40
*Gender*	.71 _(.45–1.12)_ *p* = .14	.71 _(.45–1.12)_ *p* = .14	.71 _(.45–1.12)_ *p* = .14	.71 _(.45–1.12)_ *p* = .14	.71 _(.45–1.12)_ *p* = .14	.71 _(.45–1.12)_ *p* = .14	.71 _(.45–1.12)_ *p* = .14	.71 _(.45–1.12)_ *p* = .14	.71 _(.45–1.12)_ *p* = .14
*Education*	.92 _(.71–1.20)_ *p* = .55	.92 _(.71–1.20)_ *p* = .55	.92 _(.71–1.20)_ *p* = .55	.92 _(.71–1.20)_ *p* = .55	.92 _(.71–1.20)_ *p* = .55	.92 _(.71–1.20)_ *p* = .55	.92 _(.71–1.20)_ *p* = .55	.92 _(.71–1.20)_ *p* = .55	.92 _(.71–1.20)_ *p* = .55
*Step 2*									
*Environmental smoking T1*	1.13_(.79–1.63)_ *p* = .49	1.11_(.78–1.59)_ *p = *.57	1.11_(.77–1.58)_ *p = *.58	1.23_ (.87–1.75)_ *p* = .25	1.20 _(.84–1.72)_ *p* = .32	1.27 _(.88–1.83)_ *p = *.20	1.62_(1.08–2.44)_ *p = *.02	1.63_(1.08–2.44)_ *p = *.02	1.63 _(1.08–2.46)_ *p = *.02
*Genotype*	.75_(.46–1.20)_ *p = *.23	1.35_(.83–2.19)_ *p = *.22	.97_(.59–1.61)_ *p* = .92	.71_(.44–1.16)_ *p* = .17	1.35_(.83–2.20)_ *p* = .23	1.01_(.61–1.68)_ *p = *.96	.75 _(.46–1.22)_ *p = *.25	1.38_(.84–2.24)_ *p* = .20	1.03 _(.61–1.71)_ *p* = .92
*Step 3*									
Genotype*environmental smoking	1.11 _(.55–2.25)_ *p = *.77	.73 _(.36–1.47)_ *p = *.38	.90_(.44–1.86)_ *p = *.78	.86_(.46–1.62)_ *p = *.64	1.02_(.50–2.10)_ *p = *.95	1.66_(.79–3.50)_ *p* = .18	1.07_(.42–2.72)_ *p = *.89	1.65 _(.72–3.79)_ *p = *.24	.95 _(.41–2.20)_ *p = *.91

*Note*. gender: 1 = boy, 2 = girl; OR = Odds Ratio; 95% CI = 95% Confidence Interval.

**Table 6:** Logistic regression analyses friends smoking at T1 predicting smoking onset at T5 and the moderating role of *DRD2*, *DRD4*, and *DAT1* genotypes for *study 2*.

	*Friends smoking*			*Best friends smoking*		
	*DRD2*	*DRD4*	*DAT1*	*DRD2*	*DRD4*	*DAT1*
	OR _(95% CI)_	OR _(95% CI)_	OR _(95% CI)_	OR _(95% CI)_	OR _(95% CI)_	OR _(95% CI)_
*Step 1*						
*Age*	.92 _(.75–1.12)_ *p* = .40	.92 _(.75–1.12)_ *p* = .40	.92 _(.75–1.12)_ *p* = .40	.92 _(.75–1.12)_ *p* = .40	.92 _(.75–1.12)_ *p* = .40	.92 _(.75–1.12)_ *p* = .40
*Gender*	.71 _(.45–1.12)_ *p* = .14	.71 _(.45–1.12)_ *p* = .14	.71 _(.45–1.12)_ *p* = .14	.71 _(.45–1.12)_ *p* = .14	.71 _(.45–1.12)_ *p* = .14	.71 _(.45–1.12)_ *p* = .14
*Education*	.92 _(.71–1.20)_ *p* = .55	.92 _(.71–1.20)_ *p* = .55	.92 _(.71–1.20)_ *p* = .55	.92 _(.71–1.20)_ *p* = .55	.92 _(.71–1.20)_ *p* = .55	.92 _(.71–1.20)_ *p* = .55
*Step 2*						
*Environmental smoking*	1.98 _(1.21–3.26)_ *p = *.006	2.04 _(1.24–3.37)_ *p = *.004	2.05 _(1.25–3.38)_ *p = *.004	1.05 _(.69–1.60)_ *p* = .81	1.09 _(.71–1.67)_ *p = *.70	1.06 _(.70–1.60)_ *p = *.79
*Genotype*	.74_(.39–1.19)_ *p = *.21	1.41 _(.86–2.30)_ *p = *.17	.95 _(.56–1.56)_ *p = *.80	.74_(.45–1.19)_ *p* = .21	1.33 _(.82–2.16)_ *p = *.25	.95_(.57–1.59)_ *p = *.86
*Step 3*						
Genotype* environmental smoking	.74 _(.27–2.06)_ *p = *.57	.61_(.23–1.61)_ *p = *.32	1.12 _(.43–2.92)_ *p = *.82	.60_ (.24–1.46)_ *p = *.26	.83_(.33–2.06)_ *p* = .68	.92 _(.37–2.28)_ *p = *.86

*Note*. gender: 1 = boy, 2 = girl; OR = Odds Ratio; 95% CI = 95% Confidence Interval.

### Additional analyses

Interaction effects were also examined in a multivariate analysis controlling for all environmental smoking factors (i.e., paternal and maternal smoking, sibling smoking, and (best) friend(s′) smoking). The results did not show differences compared with the separate analyses per environmental smoking factor.

Besides this, we tested whether other substance use (e.g., alcohol use) and phenomena that are known to be related to smoking (e.g., depressive feelings, loneliness) had an effect on the interplay between genes related to the dopaminergic system and environmental smoking, by controlling for different smoking related variables (*study 1*: alcohol, depressive feelings, and bullying; *study 2:* alcohol, drugs use, personality factors, depressive feelings, loneliness, self-control, self-esteem, eating behavior) in step one of the analyses. No different interaction effects were found.

## Discussion

The present study tested the interactions between environmental smoking (i.e., paternal and maternal smoking, sibling smoking, peer smoking) and the dopamine receptor genes *DRD2* and *DRD4* as well as the dopamine transporter gene *DAT1* on smoking initiation using two independent data sets of early and middle-to-late Dutch adolescents. Various environmental factors (i.e., maternal, friends, and sibling smoking) were related to adolescent smoking onset. No direct effects of any of the dopamine related genes were found, except for a small effect of the *DRD4* when controlling for sibling smoking, making it not a very robust effect. No support was found for any interaction effects.

In line with the literature, we found that environmental smoking increases the likelihood that children start smoking. *Study 1* shows that maternal and friends' smoking behavior at T1 is associated with adolescent smoking at T5. *Study 2* shows a bivariate correlation between siblings and friends smoking at T5—an effect that almost disappeared in the multivariate regression analyses because of the Bonferroni correction. The results are comparable with previous studies showing that parental smoking [2] and friends' smoking [3]–[5] are related with adolescent smoking onset. The different findings for the two samples could be due to the different age groups included. Vitaro and colleagues [41] showed that the effects of environmental smoking differ per age group. In early adolescence, both parents and friends are important, whereas during late adolescence friends tend to become more influential. However, it should be noted that certain studies have demonstrated that parents and peers are similarly important throughout adolescence [42]–[45]. In sum, environment smoking is an important factor in explaining adolescent smoking onset, although more research on the timing of parent and peer smoking is necessary.

In both study samples, no direct effects of the *DRD2* or the *DAT1* on smoking onset were found. For the *DRD4* an association was only found when controlling for sibling smoking. However, the bivariate correlation was also not significant. These results are in line with other studies showing weak effects for the *DRD2* genotype and inconsistent evidence for the effects of the *DRD4* and *DAT1* genotypes on smoking [13], [14], [18].

Previous studies have suggested that it is important to focus on the interaction between genes and the environment [7]. However, we found no interaction effects between environmental smoking and three genetic polymorphisms related to the dopaminergic system, indicating that effects of environmental smoking on smoking initiation are similar for carriers and non-carriers of those specific polymorphisms related to the dopaminergic system. It seems as if smoking initiation is primarily instigated by a variety of environmental factors—of which environmental smoking is an important one—and that genes related to the dopaminergic system do not play a direct role or indirect role via an interplay with environmental factors on smoking initiation. This is in line with behavioral genetic studies showing that shared environmental factors played a main role in smoking initiation, whereas in smoking persistence the influence of genetic factors increased [9], [22], [23]. However, it should be kept in mind that we only tested three single nucleotide polymorphisms (SNPs), whereas multiple loci might be involved in the development of initial dependence symptoms. Because of linkage disequilibrium (i.e., non-random association between alleles), genotyping several SNPs within the DRD2, DRD4 or DAT1 gene and adjacent genes would be necessary to provide insight into other associated variants. Therefore, we emphasize that our results regarding the effects of the DRD2, DRD4 and DAT1 polymorphisms must be interpreted with caution.

In this study we concentrated on potential interaction effects between genes related to the dopaminergic system and environmental smoking. Berridge and Robinson [46], [47] distinguished in their incentive salience theory between the feelings of pleasure of smoking (“liking”) and the more obsessive craving processes (“wanting”). The neural substrates of the “liking” of smoking seem to be mainly located in opioid neurotransmission, whereas “wanting” is associated with dopaminergic neurotransmissions [47], [48]. Wanting is supposed to be the result of changed (sensitized) brain systems following smoking [46], [47]. To experience the dopamine-related craving of nicotine (“wanting”), one should have smoked a sufficient number of cigarettes on more than one occasion, which might make major dopaminergic involvement in smoking onset less likely. Alternatively, the “liking” of smoking has been suggested to become less important during the transition from smoking onset to regular smoking behavior. This would suggest that opioid-related pleasure effects of smoking might be more important in smoking onset [48] and that a closer look at the interplay between genes related to the opioid system and environmental smoking would render new GxE interactions that are predictive of smoking initiation. In sum, more research on genes from this system (e.g., *OPRM1*) and other systems, such as the serotonin system (e.g., *5-HTTLPR*) [49], is needed.

In *study 2*, gene-environment correlations were found between maternal and paternal smoking T1 and *DAT1*.We have interpreted these results with caution as they were not consistent over time (i.e., only significant correlation in maternal and paternal smoking at T1, not at T2 through T5 (results not presented here)).

In addition to the several strengths of our study, including the two independent longitudinal samples, some limitations should be acknowledged. First, in both samples adolescents reported on their own smoking behavior and environmental smoking. Although previous research has shown that self-report data about smoking [50] and report about others' smoking (e.g., parental smoking [51], best friends' smoking [37]) are generally reliable, multi-informant data might have resulted in more accurate data. Second, the dopaminergic system is not only related to smoking but also related to the use of other substances and behaviors. Therefore, approaching smoking initiation as a single phenotype is limited. We controlled for all available smoking-related variables in additional analyses, and results remained unchanged. However, in future studies it would be advised to use super controls to assess the effects of dopamine related polymorphisms on smoking initiation [52]. Super controls are extensively screened to exclude a number of associated behaviors related to smoking and the use of these controls allows for stronger conclusions regarding the presence or absence of dopamine related polymorphisms on smoking initiation in youth. Third, by examining ‘simple’ genotype-phenotype associations, essential processes in gene expression that are not caused by the DNA sequence (i.e., epigenetics) are overlooked. For a full understanding of how gene-environment interactions lead to smoking initiation, epigenetic mechanisms should be taken into account [53]. Fourth, adolescents with a history of smoking at the first assessment were excluded from the analyses. Analyses showed that the genotyped smokers were more likely to have smoking sibling and friends. Therefore, mechanisms underlying smoking onset might differ for those who start early in preadolescence and those who start during adolescence. Our results could not be generalized to preadolescents or adults. However, the majority of people start smoking during adolescence [1]. Future research should study early smoking initiation among preadolescent children (i.e., 9 to 11 years old).

In conclusion, in the two independent samples we found that adolescent smoking onset is positively affected by environmental smoking. No evidence suggested a direct effect or interaction effect of *DRD2*, *DRD4*, and *DAT1* genotypes on the relationship between environmental smoking and smoking onset, indicating that carriers and non-carriers are equally affected by the smoking behavior of their environment. More studies are needed to increase the understanding of the interplay between genetics and environmental factors on adolescent smoking onset. Currently results indicate that it is important to focus on environmental smoking in smoking prevention.
